# Impact of bile acids on the growth of human cholangiocarcinoma via FXR

**DOI:** 10.1186/1756-8722-4-41

**Published:** 2011-10-12

**Authors:** Jiaqi Dai, Hongxia Wang, Yihui Shi, Ying Dong, Yinxin Zhang, Jian Wang

**Affiliations:** 1 Division of General Surgery, Shanghai Renji Hospital, Shanghai Jiao Tong University School of Medicine, Shanghai 200127, China; 2 Division of Oncology, Shanghai Renji Hospital, Shanghai Jiao Tong University School of Medicine, Shanghai 200127, China; 3 Cancer Biology, Bioscience Division, Stanford Research Institute (SRI) International, 333 Ravenswood Avenue, Menlo Park, CA 94025, USA; 4 Division of Endocrinology, Shanghai Renji Hospital, Shanghai Jiao Tong University School of Medicine, Shanghai 200127, China

## Abstract

**Background:**

The objective of the study was to investigate the effect of different types of bile acids on proliferation of cholangiocarcinoma and the potential molecular mechanisms.

**Methods:**

PCR assay and Western blot were performed to detect the expression of farnesoid × receptor (FXR) in mRNA and protein level. Immunohistochemical analysis was carried out to monitor the expression of FXR in cholangiocarcinoma tissues from 26 patients and 10 normal controls. The effects on in vivo tumor growth were also studied in nude mouse model.

**Results:**

Free bile acids induced an increased expression of FXR; on the contrary, the conjugated bile acids decreased the expression of FXR. The FXR effect has been illustrated with the use of the FXR agonist GW4064 and the FXR antagonist GS. More specifically, when the use of free bile acids combined with FXR agonist GW4064, the tumor cell inhibitory effect was even more pronounced. But adding FXR antagonist GS into the treatment attenuated the tumor inhibitory effect caused by free bile acids. Combined treatment of GS and CDCA could reverse the regulating effect of CDCA on the expression of FXR. Administration of CDCA and GW 4064 resulted in a significant inhibition of tumor growth. The inhibitory effect in combination group (CDCA plus GW 4064) was even more pronounced. Again, the conjugated bile acid-GDCA promoted the growth of tumor. We also found that FXR agonist GW4064 effectively blocked the stimulatory effect of GDCA on tumor growth. And the characteristic and difference of FXR expressions were in agreement with previous experimental results in mouse cholangiocarcinoma tissues. There was also significant difference in FXR expression between normal and tumor tissues from patients with cholangiocarcinoma.

**Conclusions:**

The imbalance of ratio of free and conjugated bile acids may play an important role in tumorigenesis of cholangiocarcinoma. FXR, a member of the nuclear receptor superfamily, may mediate the effects induced by the bile acids.

## Background

Cholangiocarcinoma is notoriously difficult to diagnose and is associated with high mortality [1]. At diagnosis, most cases become inoperable [2]. Patients with cholangiocarcinoma respond extremely poorly to the conventional chemotherapy and radiation therapy. Thus, there is an urgent need to develop effective new therapeutic strategies.

It is known that the balance of various bile acids is crucial to lipid metabolism. Significant progress has occurred in the understanding of their roles in carcinogenesis [3,4]. The "Toxic Bile" concept has been proposed to explain the effects of bile acids on cholestatic liver diseases [5]. Other than cholelithiasis, studies have also found that there is an excess risk of forming malignant tumors in some organs exposed to high concentration of bile acids, such as in the gastrointestinal tract [6]. Several epidemiologic studies have implicated that the alterations of the composition of bile acids increased the incidence of colorectal adenocarcinoma [7]. Additionally, bile acids were found to stimulate cell metaplasia in the mucosa of the stomach [8]. According to Mühlbauer's report, conjugated bile acid could stimulate NF-κB pathway and regulate the expression of inflammatory factors, leading to abnormal proliferation of epithelial cells in the colon [9]. Dvrok and Wehbe found that a mix of GCA, GCDCA, GDCA and TCA stimulated tumor growth due to chronically elevated IL-6 expression [10,11]. In view of previous studies, bile acids are thought as not only the metabolic products of lipid metabolism, but also as possible tumor regulating factors. However, there are a number of important questions yet to be answered. 1) Do changes in the human bile acid composition affect the activation of nuclear receptors and cell signaling pathways in the cholangiocarcinoma in a physiologically significant way? 2) What is the underlying molecular mechanism of various types of bile acids on the occurrence and development of cholangiocarcinoma?

Farnesoid × receptor (FXR) is a nuclear receptor for bile acids, which is very important in bile acid homeostasis, as well as in glucose and lipid metabolism [12-14]. FXR consists of two primary domains, the DNA binding domain and the ligand binding domain. Through these functional domains, extranuclear signaling can be conveyed to the specific DNA site. Recent studies have demonstrated that the function of FXR is also related to the pathological process of cholangiocarcinoma [15,16]. Yang and Kim observed spontaneous hepatic cancer and cholangiocarcinoma in FXR-null mice [17,18]. In addition, it was reported that up-regulation of FXR by its agonist could induce cell apoptosis [16,19,20]. All of the above studies strongly suggest that FXR might play a key role in the tumor occurrence and development. As reported in literature, FXR agonist could inhibit other nuclear factors which controlled cell growth, apoptosis or tumorigenesis [16].

In our previous study, we found that free bile acids caused dose- and time-dependent inhibition of cholangiocarcinoma cells, whereas the conjugated form caused dose- and time-dependent stimulation [21]. Here, we focused our research on FXR and its role in the function of bile acids in mouse and human tumor tissues, to better understand the molecular mechanisms underlying the effect of bile acids on cholangiocarcinoma.

## Methods

### 1. Cell culture and agents

Human cholangiocarcinoma cell line QBC 939 was obtained from Shanghai Tongji University (Shanghai, China). The cells were maintained in a 37°C RPMI-1640 medium (Invitrogen, CA, USA), and the culture was kept in a humidified atmosphere of 5% CO_2 _and supplemented with 10% fetal bovine serum (Biowest, Madrid, Spain). Sodium salts of free bile acids-cholic acid (CA), deoxycholic acid (DCA), chenodeoxycholic acid (CDCA), and their glycine conjugates-glycocholic acid (GCA), glycochenodeoxycholic acid (GDCA) and glycochenodeoxycholic acid (GCDCA) were purchased from Sigma Corp (St Louis, MO). GS, a FXR antagonist, was purchased from Calbiochem Corp (San Diego, CA). GW4064, a FXR agonist, was purchased from Tocris Corp. (Ellisville, MO).

Cells were cultured overnight before treatment. Next day, six different bile acids (CA 200 μmol/L; DCA 200 μmol/L; CDCA 200 μmol/L; GCA 800 μmol/L; GDCA 400 μmol/L; GCDCA 400 μmol/L) were added to the cell culture for 48 hours.

### 2. Treatment of xenograft tumor in nude mice

To form the xenograft tumors, 2 × 10^6 ^cells were injected subcutaneously into the nude mice. 14 days later, the volume of the tumors reached about 100 mm^3^. Then, 36 mice were randomized into six groups. In the control group, mice were fed with 100 μl sterile water and intraperitoneally injected with 100 μl DMSO. In the CDCA group and GDCA group, mice were fed with different bile acids (400 mg/kg) and intraperitoneally injected with 100 μl DMSO. In the GW4064 group (GW4064, a synthetic FXR agonist, Tocris Corp., UK), mice were intraperitoneally injected with GW4064 (30 mg/kg) and fed with 100 μl sterile water. In the combined treatment groups, mice were injected with GW4064 combined with feeding of CDCA or GDCA. After the treatment, all mice were kept in a lamina flow environment for another 7 days and the volume of the xenograft was recorded before and after the treatment according to the following formula: V = (length × width^2^)/2.

### 3. RNA preparation and PCR assay (RT-PCR and real-time PCR)

Total RNA was isolated using Trizol reagent (Invitrogen, CA, USA.) according to the manufacturer's recommendation. After being washed with 75% ethanol, the final RNA extracts were eluted in 20 μl distilled diethyl pyrocarbonate-treated water. The concentration and purity of RNA were measured using a spectrophotometer. The complementary DNA was synthesized according to the manufacturer's instruction (Fermentas, Canada). The PCR primers for FXR and GAPDH gene amplification were as follows: FXR's forward 5'-acaatccaaggaggtagaagac-3', reverse 5'-gaagaaatccaggaaactaagag-3'; GAPDH's forward 5'-ccctgttgctgtagccaaattc-3', reverse 5'-acccactcctccacctttga-3'. The conditions consisted of 40 cycles of denaturation at 90°C for 30 s, annealing at 55°C for 30 s and extension at 72°C for 60 s in a PTC0200 thermal cycle system (Bio-Rad, CA, USA). The RT-PCR products were examined by electrophoresis on a 2% agarose gel and quantified by grey levels measurements. The real-time PCR assay was measured in Light Cycler 480 Real-Time PCR System (F. Hoffmann-La Roche, Ltd) under the following conditions: 40 cycles of pre-denaturation at 90°C for 10 s, denaturation at 90°C for 10 s and annealing at 60°C for 15 s. The PCR primers were as follows: FXR's forward 5'-GATTGCTTTGCTGAAAGGGTC-3', reverse 5'-CAGAATGCCCAGACGGAAG-3'. β-actin's forward 5'-TTGCTGATCCACATCTGCT-3' reverse 5'-GACAGGATGCAGAAGGAGAT-3'. GAPDH was used as an internal control for RT-PCR and β-actin was used as an internal control of real-time PCR.

### 4. Western blot analysis

Proteins were extracted using a nuclear and cytoplasm extraction kit (Pierce Biotechnology, IL, USA). Western blot analysis was performed as described previously [22]. Rabbit anti human FXR antibody (Santa Cruz Biotechnology, Santa Cruz, CA) was diluted to 1:1000. Antibody binding were detected using the Odyssey Infrared Image S-120 system (Li-cor Inc, CA). TBP (Santa Cruz Biotechnology, Santa Cruz, CA) levels were used as internal controls. TBP, TATA-binding protein, a kind of nucleoprotein, served as the loading control for FXR. And we used grey level to quantify FXR expression.

### 5. Immunohistochemical analysis

After obtaining informed consent from patients and after receiving the approval of the ethics committee, a total of 26 patients with cholangiocarcinoma undergoing elective surgery were entered sequentially, into this prospective study. We also collected 10 normal bile duct tissues from donors for transplantation. Tissues obtained from the nude mice and patients were fixed in paraformaldehyde and paraffin wax for further analysis.

Immunohistochemical analysis was performed as described previously [22]. Anti FXR antibodies was diluted to 1:50. Every slide was reviewed by two pathologists via double blind observation procedures. Immunostaining scores were calculated based on the percentage of immunostained cells and the intensity score. Immunostaining intensity scores were calculated by using the following formula: weighted signal intensity = percentage of immunostained cells × average intensity score. The definition for the calculated scores is as following: 0 point as negative staining; 1~2 points as slight staining (+); 3~4 points as moderate staining (++); 5~6 points as strong staining (+++). We also used Zeiss system to analyze positive rate and made a histogram to describe the difference between control group and treated groups.

### 6. Statistical analysis

Data were shown as mean values ± S.E. and SAS 8.0 software (SAS Institute, USA) was used for statistical analysis. Student's t-test (two-tail) was used throughout the present study and Kruskal-Wills test was used to analyze the expression of FXR in cholangiocarcinoma and normal tissues. The differences were considered statistically significant for p < 0.05.

## Results and Discussion

**Figure 1:**
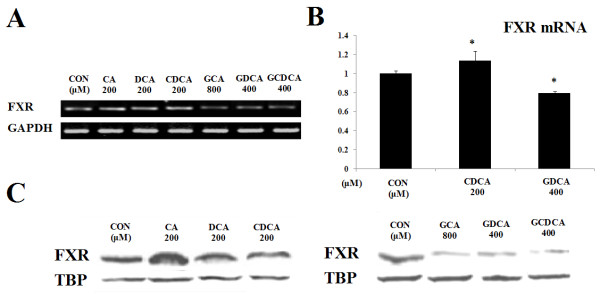
**Bile acids regulated the expression of FXR**. A, RT-PCR analysis showed that free bile acids (CA, DCA, CDCA) enhanced the mRNA expression of FXR whereas conjugated bile acids (GCA, GDCA, GCDCA) decreased FXR mRNA level. GAPDH served as the loading control. B, Realtime-PCR analysis showed that differences of FXR mRNA expression according to density level after CDCA and GDCA treatment. β-actin served as an internal control. C, The protein expression of FXR was tested by Western blot. Free bile acids increased the expression of FXR, while conjugated bile acids decreased the expression of the FXR protein. TBP (TATA-binding protein, a kind of nucleoprotein) served as the loading control.

To determine whether FXR expression is responsible for the changes in cell viability and apoptosis, we used regular RT-PCR and real-time PCR to detect the mRNA expression of FXR and Western blot to compare the protein expression of FXR. Our results showed that the expression of FXR was altered by different bile acids. The free bile acids (CA, DCA and CDCA) induced an increase in the mRNA (Figure 1A,B) and protein expression (Figure 1C) of FXR. On the contrary, treatment by the conjugated bile acids (GCA, GDCA and GCDCA) decreased the mRNA and protein expression of FXR. According to density level, the mRNA expression of FXR was detected as 1.16 folds (200 μM CA group), 1.12 folds (200 μM DCA group), 1.16 folds (200 μM CDCA group), 0.60 folds (800 μM GCA group), 0.61 folds (400 μM GDCA group) and 0.61 folds (400 μM GCDCA group), compared with the control group. In the part of real-time PCR assay, it indicated that CDCA increased mRNA expression as 1.14 folds of control, while GDCA decreased the expression of FXR mRNA as 0.79 folds of control. And the protein expression of FXR was 1.56 folds (200 μM CA group), 1.24 folds (200 μM DCA group), 1.25 folds (400 μM CDCA group), 0.23 folds (800 μM GCA group), 0.23 folds (400 μM GDCA group) and 0.35 folds (400 μM GCDCA group). The results indicated that bile acid regulated the cell growth through its physiological receptor FXR, which has been reported to be related to the signal transduction of cell growth [23-26]. FXR is a member of the nuclear receptor superfamily, which contains thyroid hormones, steroid hormones, retinoid and orphan nuclear receptors. FXR can bind to specific DNA sequences and activate gene transcription by forming a heterodimer with retinoid × receptor. Besides biliary cancer, FXR has also been implicated and associated with breast cancer [26] and colon cancer [27]. In contrast to its well-established mechanism in regulating bile acid homeostasis, little is known about how FXR functions in carcinogenesis. In our experiment, besides the changes in cell viability and apoptosis caused by different bile acids, changes in the expression of the bile acid receptor FXR were also observed. We also found that the alteration of FXR expression was inversely proportional to the change of cell growth. These results suggest that FXR is an important signaling receptor when cells are treated with bile acids. We speculate that the tumorigenesis may initiate due to decreased FXR expression. This is consistent with previous study findings that deficiency of FXR could lead to cell oxidative stress injury and hyperplasia [28].

**Figure 2:**
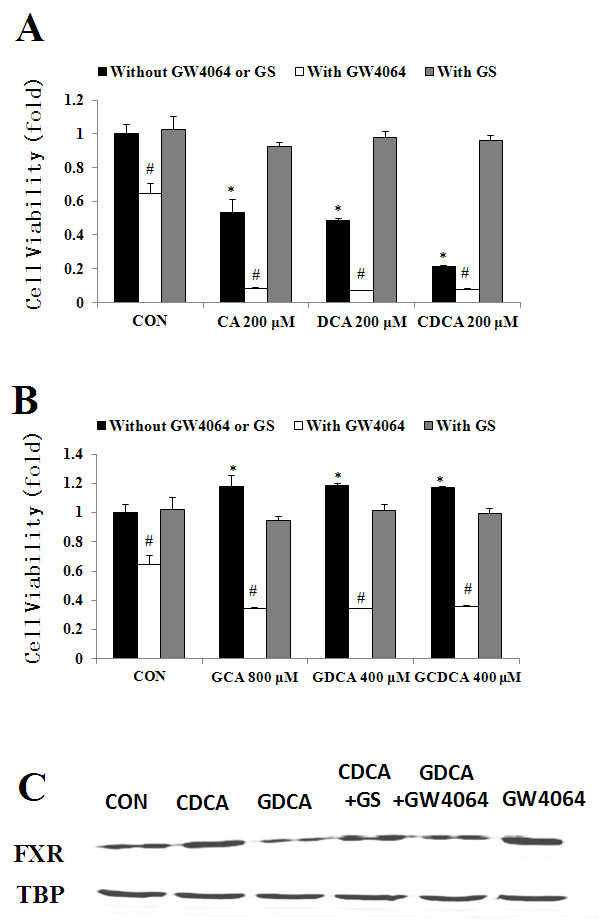
**FXR antagonist/agonist changed the effects of bile acids on FXR expression**. A, combination of GS (FXR antagonist) or GW 4064 (FXR agonist) with free bile acids. B, combination of GS or GW 4064 with conjugated bile acids. C, bile acids and FXR antagonist/agonist changed the expression of FXR. TBP: TATA-binding protein (loading control). * p < 0.05, compared with the control group. *# p < 0.05*, compared with bile acid treatment groups.

Since the expression of FXR is closely related to cancer cell growth, interventions to manipulate FXR levels may eventually benefit patients with cholangiocarcinoma. As shown in Figure 2A, free bile acids CA, DCA and CDCA significantly reduced cell proliferation of cholangiocarcinoma cells, the inhibition rate was 46.7% (CA 200 μmol/L), 51.5% (DCA 200 μmol/L) and 78.5% (CDCA 200 μmol/L) respectively (p < 0.05, compared with the control group). When combined with GW4064, the inhibitory effect of the free bile acids on cell growth was even more pronounced (inhibition rate: 91.8%, 93.1% and 92.3%, respectively). On the contrary, adding GS into the treatment attenuated the inhibitory effect caused by free bile acids. The inhibition rate in GS and free bile acid combination group was reduced to 7.74%, 2.41% and 3.73%, respectively. Conjugated bile acids enhanced cholangiocarcinoma cell growth significantly (Figure 2B). However, GW4064 reversed the effect of conjugated bile acids on QBC 939 cells. When the conjugated bile acids were combined with GW4064, cell viability was significantly decreased (inhibition rate: 65.7%, 65.6% and 64.1%, p < 0.05). When the conjugated bile acids were combined with GS, the stimulatory effect of the conjugated bile acids on cell growth was abrogated.

We next used FXR antagonist or agonist to co-treat with either free or conjugated bile acids, and detected the protein expression of FXR. As shown in Figure 2C, free bile acids increased the expression level of FXR. Combined treatments of GS and CDCA could reverse the regulating effect of CDCA on the expression of FXR. Additionally, when treating cells with conjugated bile acids-GDCA, FXR expression was decreased. When treating cells with both FXR agonist-GW4064 and conjugated bile acid-GDCA, the effect caused by GDCA alone was reversed.

**Figure 3:**
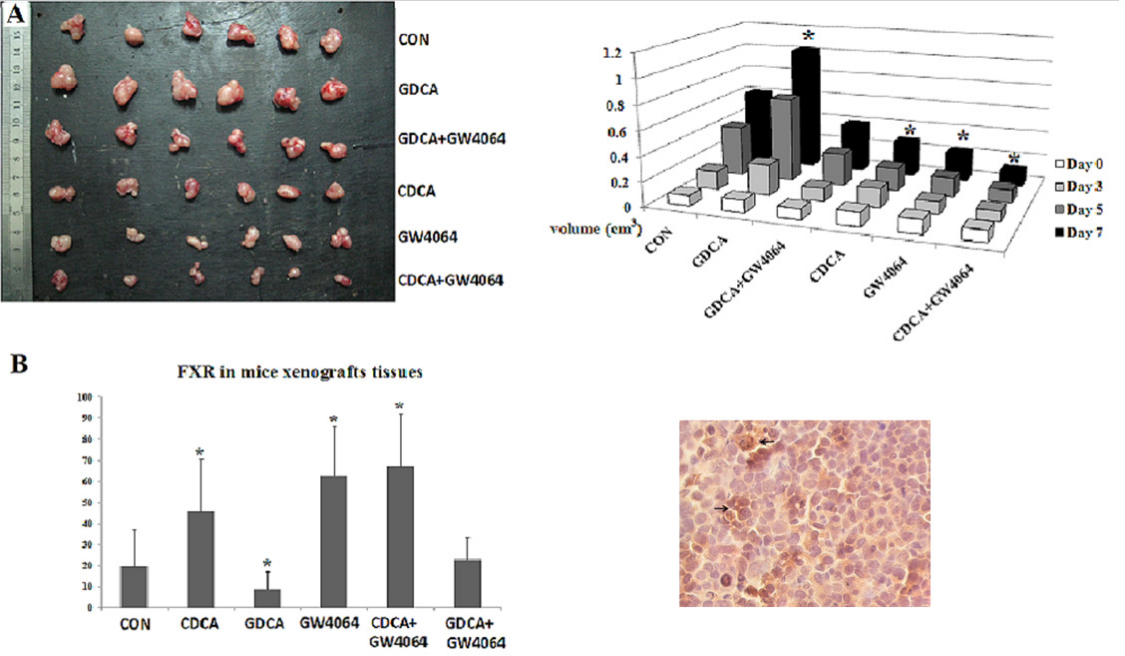
**Effect of bile acids and GW4064 (FXR agonist) on tumor growth in vivo**. A, effect of bile acids and GW4064 on tumor growth in vivo. The mean volumes of tumor in CDCA and GW 4064 were much less than those of CON group, GDCA group and GDCA+GW 4064 group due to the enhancement of FXR expression. The inhibitory effect in CDCA plus GW 4064 group was even more pronounced. (* p < 0.05). Left panel: tumors at the end of the experiment; right panel: quantitative measures of the tumor volumes. B, Immunohistochemical staining for FXR expression in mice xenograft tissues. Right panel: quantitations of FXR expression in different groups; left panel: representative immunostaining of FXR expression in the tumor tissues. Black arrows point to positive cells.

To further assess the results in vivo, we used a nude mouse model to determine the effect of bile acids on cholangiocarcinoma (Figure 3A). Tumor volume at the end of the experiment was 0.279 ± 0.068 cm^3 ^and 0.228 ± 0.116 cm^3 ^(n = 6) in animals receiving CDCA and GW 4064, respectively. Administration of CDCA and GW 4064 resulted in a significant (*P *< 0.05) inhibition of tumor growth in both sets of animals compared with control group (tumor volume: 0.609 ± 0.089 cm^3^). The inhibitory effect in combination group (CDCA plus GW 4064) was even more pronounced when compared with control group (tumor volume: 0.120 ± 0.046 cm^3 ^vs. 0.609 ± 0.089 cm^3^, respectively; *P *< 0.05). Again, the conjugated bile acid-GDCA promoted the growth of tumor (tumor volume: 1.021 ± 0.272 cm^3^; p < 0.05, compared with the control group). In addition, we found that FXR agonist GW4064 effectively blocked the stimulatory effect of GDCA on tumor growth. The volumes of tumor xenografts in the combination of GDCA and GW4064 group were similar to data in the control group (Table 1).

**Table 1:** The Change of Tumor Volume before and after Treatment

Group	Volume D0 (cm^3^)	Volume D3 (cm^3^)	Volume D5 (cm^3^)	Volume D7 (cm^3^)	Volume Change (cm^3^)
CON	0.086 ± 0.015	0.149 ± 0.022	0.407 ± 0.056	0.609 ± 0.089	0.523 ± 0.083
GDCA	0.102 ± 0.024	0.250 ± 0.065	0.688 ± 0.176	1.021 ± 0.272	0.919 ± 0.282*
GDCA+GW4064	0.086 ± 0.046	0.106 ± 0.063	0.261 ± 0.188	0.393 ± 0.287	0.307 ± 0.260
CDCA	0.114 ± 0.028	0.155 ± 0.040	0.187 ± 0.046	0.279 ± 0.068	0.164 ± 0.080*
GW4064	0.115 ± 0.023	0.101 ± 0.035	0.154 ± 0.078	0.228 ± 0.116	0.113 ± 0.130*
CDCA+GW4064	0.092 ± 0.031	0.081 ± 0.031	0.104 ± 0.024	0.120 ± 0.046	0.027 ± 0.054*

*p < 0.05, the treatment group compared with the control group. D: day

We also performed immunohistochemistry staining to determine the degree of FXR expression in mouse cholangiocarcinoma tissue. We found the similar results of FXR expressions. As shown in Figure 3B, there were 19.76% cells stained in control group. The positive rate in CDCA treatment group was 46.09% and 62.64% in GW4064 treatment group. CDCA and GW4064 worked synergistically on the expression of FXR, because the positive rate increased to 67.09%. The results also revealed that GDCA reduced the expression of FXR to 8.80%, but GW4064 reversed the effect (the positive rate was 23.00%).

**Figure 4:**
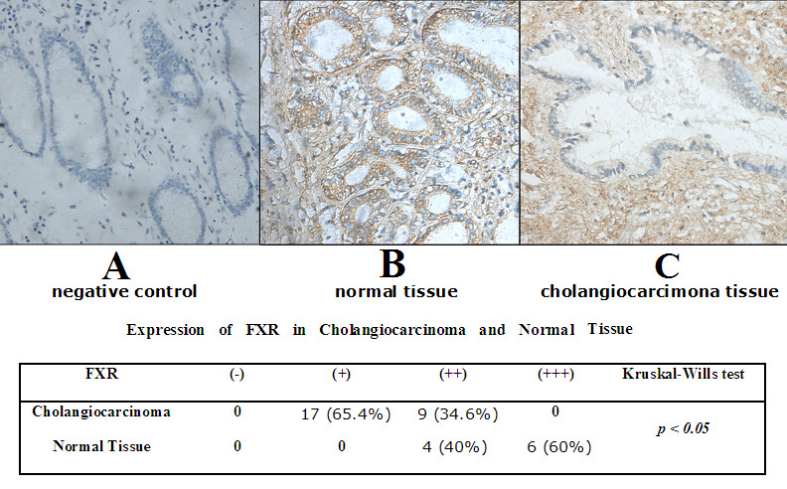
**Expression of FXR in cholangiocarcinoma tissues from patients**. Tissues from 26 patients and 10 normal controls were obtained. Immunostaining for FXR expression was done. A, negative control for immunostaining. B, FXR expression in normal biliary ducts. C, FXR expression in cholangiocarcinoma tissues. The FXR expression was quantitated according to staining intensity and reported as mild (+), moderate (++) and strong (+++), respectively. The results were summarized in the table.

Finally, we examined the expression of FXR in cholangiocarcinoma tissues from 26 patients and 10 controls. As shown in Figure 4, FXR expression was mild in 65.4% and moderate in 34.6% of the tumor tissues (Figure 4C). However, FXR was expressed strongly in 60% and moderately in 40% of the normal tissues (Figure 4B). There was strong staining of FXR in the nucleus of the normal bile duct, which reflected the high expression of FXR in normal tissue. However, in cholangiocarcinoma tissue, few cells had the nuclear staining for FXR, reflecting the reduced expression of FXR in tumor tissue. Result of Kruskal-Wills test indicates significant statistical difference of FXR expression between normal and tumor tissues. These results indicate that FXR could be a protective factor for tumor development.

We are working on over-expressing FXR in our laboratory and hope to develop a new therapeutic strategy for cholangiocarcinoma. Furthermore, our preliminary studies have shown that there are intriguing linkages between FXR and NF-kappa B pathway (data not shown). In the human cholangiocarcinoma tissues, we have not only found the decrease of FXR expression, but also noticed an unusual increase of p-IkB. On the opposite, p-IkB was very rarely observed in normal tissue. Because the level of p-IkB can reflect indirectly the activation of NF-kappa B pathway, we therefore propose that the effect of bile acids on tumor growth is related to NF-kappa B pathway. Novel agents are being developed for cancer therapy [29-32]. We believe that study of FXR expression on bile acid signaling pathway could be useful for development of novel therapy for cholangiocarcinoma.

## Conclusions

The imbalance of free and conjugated bile acids ratio may play an important role in tumorigenesis of cholangiocarcinoma. FXR, a member of the nuclear receptor superfamily, may also mediate the effects induced by the bile acids. Regulating the balance of free/conjugated bile acids as well as activating/inhibiting FXR might provide promising therapeutic approaches to treating cholangiocarcinoma patients.

## List of abbreviations

CA: cholic acid; DCA: deoxycholic acid; CDCA: chenodeoxycholic acid; GCA: glycocholic acid; GDCA: glycochenodeoxycholic acid; GCDCA: glycochenodeoxycholic acid; FXR: farnesoid × receptor; GS: guggulsterone.

## Competing interests

The authors declare that they have no competing interests.

## Authors' contributions

JW was involved in experimental designs, data acquisition and analysis data interpretation as well as manuscript preparation. JQD, YD and YXZ conducted experiments, data acquisition and interpretation of data. YHS and HXW were involved in the analysis and interpretation of data as well as manuscript preparation. All authors read and approved the manuscript.

## References

[B1] Javle Milind Hsueh Chung-Tsen Recent advances in gastrointestinal oncology - updates and insights from the 2009 annual meeting of the American Society of Clinical Oncology Journal of Hematology & Oncology 2010 3 11 21 10.1186/1756-8722-3-11 20331897 PMC2856525

[B2] Sirica AE Cholangiocarcinoma: molecular targeting strategies for chemoprevention and therapy Hepatology 2005 41 5 15 10.1002/hep.20537 15690474

[B3] Burnat G Rau T Elshimi E Hahn EG Konturek PC Bile acids induce overexpression of homeobox gene CDX-2 and vascular endothelial growth factor (VEGF) in human Barrett's esophageal mucosa and adenocarcinoma cell line Scand J Gastroenterol 2007 42 1460 1465 10.1080/00365520701452209 17852856

[B4] Schramm G Surmann EM Wiesberg S Oswald M Reinelt G Eils R König R Analyzing the regulation of metabolic pathways in human breast cancer BMC Med Genomics 2010 3 39 10.1186/1755-8794-3-39 20831783 PMC2945993

[B5] Trauner M Fickert P Halilbasic E Moustafa T Lessons from the toxic bile concept for the pathogenesis and treatment of cholestatic liver diseases Wien Med Wochenschr 2008 158 542 548 10.1007/s10354-008-0592-1 18998069

[B6] Bernstein H Bernstein C Payne CM Dvorak K Bile acids as endogenous etiologic agents in gastrointestinal cancer World J Gastroenterol 2009 15 3329 3340 10.3748/wjg.15.3329 19610133 PMC2712893

[B7] Debruyne PR Bruyneel EA Karaguni IM Li X Flatau G Müller O Zimber A Gespach C Mareel MM Bile acids stimulate invasion and haptotaxis in human colorectal cancer cells through activation of multiple oncogenic signalling pathways Oncogene 2002 21 6740 6750 10.1038/sj.onc.1205729 12360401

[B8] Park MJ Kim KH Kim HY Kim K Cheong J Bile acid induces expression of COX-2 through the homeodomain transcription factor CDX1 and orphan nuclear receptor SHP in human gastric cancer cells Carcinogenesis 2008 29 2385 2393 10.1093/carcin/bgn207 18775915

[B9] Mühlbauer M Allard B Bosserhoff AK Kiessling S Herfarth H Rogler G Schölmerich J Jobin C Hellerbrand C Differential effects of deoxycholic acid and taurodeoxycholic acid on NF-kappa B signal transduction and IL-8 gene expression in colonic epithelial cells Am J Physiol Gastrointest Liver Physiol 2004 286 G1000 1008 10.1152/ajpgi.00338.2003 14726307

[B10] Dvorak K Chavarria M Payne CM Ramsey L Crowley-Weber C Dvorakova B Dvorak B Bernstein H Holubec H Sampliner RE Bernstein C Prasad A Green SB Garewal H Activation of the interleukin-6/STAT3 antiapoptotic pathway in esophageal cells by bile acids and low pH: relevance to Barrett's esophagus Clin Cancer Res 2007 13 5305 5313 10.1158/1078-0432.CCR-07-0483 17875759

[B11] Wehbe H Henson R Meng F Mize-Berge J Patel T Interleukin-6 contributes to growth in cholangiocarcinoma cells by aberrant promoter methylation and gene expression Cancer Res 2006 66 10517 10524 10.1158/0008-5472.CAN-06-2130 17079474

[B12] Wang H Chen J Hollister K Sowers LC Forman BM Endogenous bile acids are ligands for the nuclear receptor FXR/BAR Mol Cell 1999 3 543 553 10360171 10.1016/s1097-2765(00)80348-2

[B13] Kim I Ahn SH Inagaki T Choi M Ito S Guo GL Kliewer SA Gonzalez FJ Differential regulation of bile acid homeostasis by the farnesoid × receptor in liver and intestine J Lipid Res 2007 48 2664 2672 10.1194/jlr.M700330-JLR200 17720959

[B14] Zhang Y Lee FY Barrera G Lee H Vales C Gonzalez FJ Willson TM Edwards PA Activation of the nuclear receptor FXR improves hyperglycemia and hyperlipidemia in diabetic mice Proc Natl Acad Sci USA 2006 103 1006 1011 10.1073/pnas.0506982103 16410358 PMC1347977

[B15] Trauner M The nuclear bile acid receptor FXR as a novel therapeutic target in cholestatic liver diseases: hype or hope? Hepatology 2004 40 260 263 10.1002/hep.20294 15239110

[B16] Wang YD Chen WD Wang M Yu D Forman BM Huang W Farnesoid × receptor antagonizes nuclear factor kappaB in hepatic inflammatory response Hepatology 2008 48 1632 1643 10.1002/hep.22519 18972444 PMC3056574

[B17] Yang F Huang X Yi T Yen Y Moore DD Huang W Spontaneous development of liver tumors in the absence of the bile acid receptor farnesoid × receptor Cancer Res 2007 67 863 867 10.1158/0008-5472.CAN-06-1078 17283114

[B18] Kim I Morimura K Shah Y Yang Q Ward JM Gonzalez FJ Spontaneous hepatocarcinogenesis in farnesoid × receptor-null mice Carcinogenesis 2007 28 940 946 17183066 10.1093/carcin/bgl249 PMC1858639

[B19] Huang L Zhao A Lew JL Zhang T Hrywna Y Thompson JR de Pedro N Royo I Blevins RA Peláez F Wright SD Cui J Farnesoid × receptor activates transcription of the phospholipid pump MDR3 J Biol Chem 2003 278 51085 51090 10.1074/jbc.M308321200 14527955

[B20] Modica S Murzilli S Salvatore L Schmidt DR Moschetta A Nuclear bile acid receptor FXR protects against intestinal tumorigenesis Cancer Res 2008 68 9589 9594 10.1158/0008-5472.CAN-08-1791 19047134

[B21] Wang J Dai JQ Zhang CF Effects of bile acids on expression of interleukin-6 and cell viability in QBC939 cell line Zhonghua Wai Ke Za Zhi 2010 48 919 23 21055228

[B22] Wang J Shi YQ Yi J Ye S Wang LM Xu YP He M Kong XM Suppression of growth of pancreatic cancer cell and expression of vascular endothelial growth factor by gene silencing with RNA interference J Dig Dis 2008 9 228 37 10.1111/j.1751-2980.2008.00352.x 18959596

[B23] Journe F Laurent G Chaboteaux C Nonclercq D Durbecq V Larsimont D Body JJ Farnesol, a mevalonate pathway intermediate, stimulates MCF-7 breast cancer cell growth through farnesoid × receptor-mediated estrogen receptor activation Breast Cancer Res Treat 2008 107 49 61 17333335 10.1007/s10549-007-9535-6

[B24] Wolf G Retinoic acid as cause of cell proliferation or cell growth inhibition depending on activation of one of two different nuclear receptors Nutr Rev 2008 66 55 59 10.1111/j.1753-4887.2007.00006.x 18254885

[B25] Stauber RH Wünsch D Knauer SK Fetz V An update on the pathobiological relevance of nuclear receptors for cancers of the head and neck Histol Histopathol 2010 25 1093 1104 20552557 10.14670/HH-25.1093

[B26] Swales KE Korbonits M Carpenter R Walsh DT Warner TD Bishop-Bailey D The farnesoid × receptor is expressed in breast cancer and regulates apoptosis and aromatase expression Cancer Res 2006 66 10120 10126 10.1158/0008-5472.CAN-06-2399 17047076

[B27] Modica S Murzilli S Salvatore L Schmidt DR Moschetta A Nuclear bile acid receptor FXR protects against intestinal tumorigenesis Cancer Res 2008 68 9589 9594 10.1158/0008-5472.CAN-08-1791 19047134

[B28] Li YT Swales KE Thomas GJ Warner TD Bishop-Bailey D Farnesoid × receptor ligands inhibit vascular smooth muscle cell inflammation and migration Arterioscler Thromb Vasc Biol 2007 27 2606 2611 10.1161/ATVBAHA.107.152694 18029909

[B29] Frémin Christophe Meloche Sylvain From basic research to clinical development of MEK1/2 inhibitors for cancer therapy Journal of Hematology & Oncology 2010 3 8 10.1186/1756-8722-3-8 20149254 PMC2830959

[B30] Yuan RuiRong Kay Andrea Berg William J Lebwohl David Targeting tumorigenesis: development and use of mTOR inhibitors in cancer therapy Journal of Hematology & Oncology 2009 2 45 56 10.1186/1756-8722-2-45 19860903 PMC2775749

[B31] Budhu Anuradha Ji Junfang Wang Xin W The clinical potential of microRNAs Journal of Hematology & Oncology 2010 3 37 10.1186/1756-8722-3-37 20925959 PMC2958878

[B32] Zhang Xing J Ye Hua Zeng Cheng W He Bo Zhang Hua Chen Yue Q Dysregulation of miR-15a and miR-214 in human pancreatic cancer Journal of Hematology & Oncology 2010 3 46 10.1186/1756-8722-3-46 21106054 PMC3002909

